# Trends and Clinical Characteristics of HIV and Cerebrovascular Disease in Low- and Middle-Income Countries (LMICs) Between 1990 and 2021

**DOI:** 10.1007/s11904-022-00627-9

**Published:** 2022-10-20

**Authors:** George Ransley, Stanley Zimba, Yohane Gadama, Deanna Saylor, Laura Benjamin

**Affiliations:** 1 National Hospital for Neurology and Neurosurgery, University College London Foundation Trust, London, UK; 2 Department of Internal Medicine, University Teaching Hospital, Lusaka, Zambia; 3 Division of Neurology, Faculty of Medicine and Health Sciences, Stellenbosch University, Stellenbosch, South Africa; 4 Malawi-Liverpool Wellcome Trust Clinical Research Program, Kamuzu University of Health Sciences (KUHeS), Blantyre, Malawi; 5 Department of Neurology, Johns Hopkins University School of Medicine, Baltimore, MD USA; 6 MRC LMCB, University College London, Gower Street, London, WC1E 6BT UK

**Keywords:** HIV, ART, Cerebrovascular disease, Stroke, Cognitive impairment

## Abstract

**Purpose of the Review:**

To describe trends and clinical characteristics of HIV and cerebrovascular disease between 1990 and 2021 in LMICs and identify the gaps in our understanding.

**Recent Findings:**

In the era of antiretroviral therapy (ART), people living with HIV (PLWH) live longer and risk excess cerebrovascular events due to ageing and HIV-driven factors. Despite the highest burden of HIV infection in low-to-middle income countries, there is underreporting in the literature of cerebrovascular events in this population. We systematically reviewed published literature for primary clinical studies in adult PLWH and cerebrovascular disease in LMICs.

**Summary:**

The clinical phenotype of cerebrovascular disease among PLWH over the last three decades in LMICs has evolved and transitioned to an older group with overlapping cerebrovascular risk factors. There is an important need to increase research in this population and standardise reporting to facilitate understanding, guide development of appropriate interventions, and evaluate their impact.

## Introduction

The global roll-out of effective antiretroviral treatment (ART) regimens for people living with HIV (PLWH) has significantly improved life expectancy [1, 2]. As this population ages, it has been observed that other comorbidities such as cerebrovascular disease (CVD) are seen with greater frequency than in the general population [3, 4••, 5, 6, 7••]. CVD can manifest as stroke (affecting large-to-medium sized arterial vessels) or cognitive impairment (among stroke populations, or non-stroke populations with disease of the small vessels leading to lacunar infarcts or microbleeds). On average, 40–50% of survivors of stroke develop some form of cognitive dysfunction, suggesting that vascular cognitive impairment could become the most common precursor to dementia [8].

There are likely to be multiple mechanisms underlying CVD in PLWH, including HIV-associated factors (chronic inflammation, vasculopathy, opportunistic infections, cardioembolism and coagulopathy) [9], in interplay with traditional cardiovascular risk factors, which may be accelerated by HIV infection or occur through the normal ageing process. In addition, some ARTs have an additive role; for example, specific protease inhibitors are associated with hypercholesterolaemia which, in turn, increases CVD risk [4••, 10].

Much of the work elucidating the associations between HIV and CVD has been carried out in populations in high-income countries (HICs). However, the global burden of HIV is centred in low- and middle-income countries (LMICs) [11], with 12% of the global population, seeing 71% of global HIV infection [12]. This is especially true for Sub-Saharan Africa (SSA). Though the mechanisms driving CVD in PLWH in LMICs and HICs overlap, there will likely be significant regional and cultural variations. As examples, hypertension prevalence is higher in LMICs than HICs and there is evidence that this gap is widening [13]. Other CVD risk factors such as drug and alcohol use vary greatly between populations; and populations in LMICs generally have comparatively decreased access to healthcare resources than their HIC counterparts. Aetiopathogenesis of CVD in PLWH must therefore be studied in LMICs specifically.

Previous reviews have highlighted the paucity of evidence on CVD in PLWH in LMICs [14, 15]. This, coupled with the changing epidemiology of stroke in PLWH, and evidence that stroke in PLWH affects younger individuals, is of greater severity and has greater mortality [15] compared to HIV-uninfected populations, must prompt further study if the often already fragile health systems serving these populations are to adapt to and cope with this rising tide of disease.

This systematic review aims to describe trends and clinical characteristics of HIV and CVD between 1990 and 2021 in LMICs and identify the gaps in our understanding which require further elucidation.

## Methods

### Search Strategy and Selection Criteria

**Fig. 1:**
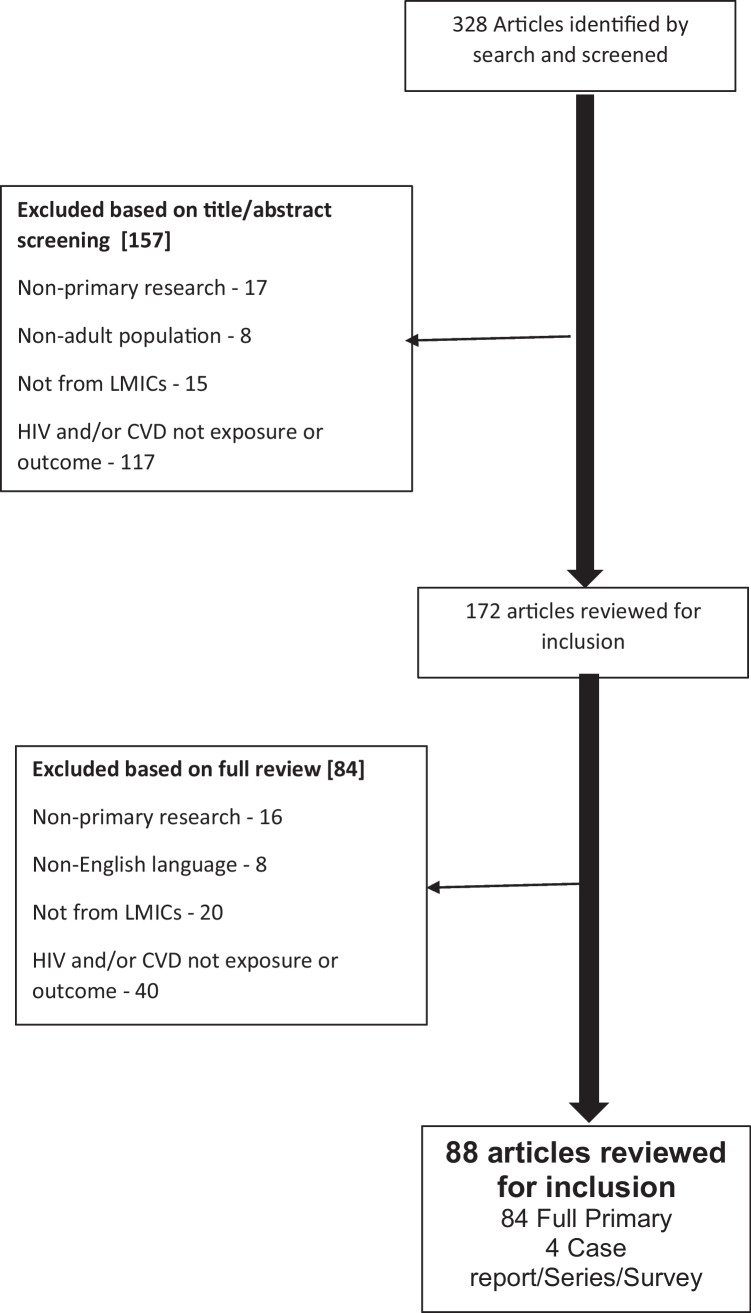
Flow diagram of search results and study inclusion and exclusion at each stage of the methods

We identified references for this review by searching Medline and PubMed for articles published in English between Jan 1990 and Dec 2021 using the terms, ‘cerebrovascular disorders’ OR ‘stroke’ OR ‘intracranial arteriosclerosis’ OR ‘arteriosclerosis’ OR ‘intracranial embolism’ OR ‘subarachnoid haemorrhage’ OR ‘intracranial haemorrhage’ OR ‘cerebral haemorrhage’ OR ‘vascular disease’ OR ‘vasculitis’ OR ‘CNS vasculitis’ OR ‘vasculopathy’ OR ‘atherosclerosis’ OR ‘cerebral venous thrombosis’ AND ‘human immunodeficiency virus’ OR ‘HIV’ OR ‘ART’ OR ‘PLWH’ AND ‘Asia’ OR ‘South America’ OR ‘Africa’ OR ‘Subsaharan Africa’ OR ‘developing country(ies)’ OR ‘least developed country(ies)’ OR ‘least developed nation(s)’ OR ‘under-developed nation(s)’ OR ‘third world nation(s)’ OR ‘third-world country(ies)’ OR ‘less-developed nation’ OR ‘underdeveloped country’ [term exploded]. The addition of the term ‘AIDS’ did not yield any further publications. Articles were also identified through the reference lists of selected publications and a search of the Cochrane Database. Only articles published in English were included. We excluded reviews if they did not report new primary data, studies limited purely to comparisons of diagnostic techniques, studies on non-cerebrovascular manifestations of HIV, and studies that did not report primary data from an LMIC. LMIC is defined by the world bank as those with <$1085 up to $13205 in per capita gross national income. The inclusion of case reports was limited to Fig. 1.

### Data Extraction

Titles and abstracts of each identified manuscript were initially screened for eligibility. Reason for exclusion was recorded for all references not meeting inclusion criteria. Full text manuscripts were then reviewed by a single author for those references thought to meet inclusion criteria based on screening of the title and abstract. For each reference meeting inclusion criteria, we extracted data for the following categories: (1) study year (s) and sites involved, (2) study design, (3) participants number and clinical characteristics, (4) HIV factors [e.g. stage of disease and ART use], (5) comorbidities, (6) aetiology of ischaemic stroke using the Trial of ORG 10172 in Acute Stroke Treatment (TOAST) classification or variations of this, and (5) clinical outcomes such as mortality, severity (e.g. National Institutes of Health Stroke Scale (NIHSS)), and functional status (e.g. modified Rankin Scale (mRS)). Data were abstracted into a pre-specified Microsoft Excel spreadsheet.

## Results

Our search returned 329 articles, of which 157 were excluded through screening of the title and abstract, and a further 84 were excluded based on a full-text review of the manuscripts (Fig. 1). Articles were excluded because they were not primary research (*n* = 33), included non-adult populations (*n* = 8), were in a language other than English (*n* = 8), did not originate from LMICs (*n* = 44), or because HIV and/or cerebrovascular disease was not an exposure or outcome (*n* = 157). After exclusion, 88 articles were included in the review.

Characteristics of included studies are shown in Table 1. Cross-sectional studies (*n* = 44, 51%) constituted the majority of included studies, followed by cohort studies (*n* = 24, 28%), case–control studies (*n* = 13, 15%), case series (*n* = 3, 3%), case reports (*n* = 1) and mixed methods studies (*n* = 1). Cross-sectional studies ranged from 25 to ~ 42,000 participants with a median sample size of 238, while cohort studies ranged from 26 to ~ 30,000 with a median sample size of 320.

**Table 1:** Characteristics of all included studies

Year	First author	Reference no	Country	Continent	Study design	Sample size
1992	Perriëns, JH	[57]	Democratic Republic of Congo	Africa	Cross-Sectional	104
2000	Hoffmann, M	[20]	South Africa	Africa	Cohort	320
2000	Hoffmann, M	[58]	South Africa	Africa	Case–Control	1,298
2003	Mochan, A	[16]	South Africa	Africa	Case Series	35
2004	Connor, M	[59]	South Africa	Africa	Cross-Sectional	42,378
2005	Deshpande, A	[28]	India	Asia	Cross-Sectional	300
2005	Patel, V	[60]	South Africa	Africa	Cohort	293
2005	Otedo	[18]	Kenya	Africa	Case Series	8
2005	Kumwenda, JJ	[30]	Malawi	Africa	Cohort	98
2006	Joshi, R	[61]	India	Asia	Cross-Sectional	1,354
2007	Jowi, J	[62]	Kenya	Africa	Cross-Sectional	150
2007	Tipping, B	[29]	South Africa	Africa	Cohort	1,087
2008	Jowi, JO	[63]	Kenya	Africa	Cross-Sectional	2,629
2008	Andrade, ACO	[64]	Brazil	South America	Cross-Sectional	69
2008	Onwuchekwa, AC	[65]	Nigeria	Africa	Cross-Sectional	54
2009	Robbs, J	[17]	South Africa	Africa	Case Series	226
2009	Silva, EFR	[66]	Brazil	South America	Mixed: Case–Control and Cross-Sectional	319
2009	Heikinheimo, T	[36]	Malawi	Africa	Cohort	147
2010	Maduagwu, S	[67]	Nigeria	Africa	Cross-Sectional	236
2010	Maier, D	[68]	Tanzania	Africa	Cross-Sectional	3,238
2011	Fourie	[69]	South Africa	Africa	Case–Control	600
2011	Benjamin Longo-Mbenza	[70]	DRC	Africa	Cross-Sectional	116
2011	Neto, JPM	[71]	Brazil	South America	Cohort	1,415
2012	Lee, B	[41]	Thailand	Asia	Case–Control	111
2012	Falcão, M	[72]	Brazil	South America	Cross-Sectional	122
2012	Owolabi, L	[73]	Nigeria	Africa	Cohort	71
2013	Ngatchou	[74]	Cameroon	Africa	Case–Control	204
2014	Ssinabulya, I	[75]	Uganda	Africa	Cross-Sectional	245
2014	Mossong, J	[76]	South Africa	Africa	Cross-Sectional	11,497
2015	Smit, M	[77]	Zimbabwe	Africa	Cohort	Statistical modelling
2015	Nakibuuka	[78]	Uganda	Africa	Cross-Sectional	5,481
2015	Schoffelen	[79]	South Africa	Africa	Cross-Sectional	906
2015	Pacheco, AG	[80]	Brazil	South America	Cohort	649
2015	Asiki, G	[81]	Uganda	Africa	Cohort	163
2015	Zimba, S	[81]	Zambia	Africa	Case–Control	104
2015	Fourie, CM	[83]	South Africa	Africa	Case–Control	309
2015	Valenzuela-Rodríguez, G	[84]	Peru	South America	Cohort	26
2015	Heikinheimo, T	[85]	Malawi	Africa	Cross-Sectional	25
2016	Benjamin, L	[7]	Malawi	Africa	Case–Control	725
2016	Pacheco, A	[86]	Brazil	South America	Cohort	15,860
2016	Mapoure, Y	[24]	Cameroon	Africa	Cohort	407
2016	Okeng'o	[87]	Tanzania	Africa	Cohort	224
2016	Divala, OH	[88]	Malawi	Africa	Cross-Sectional	952
2016	Osegbe, I	[89]	Nigeria	Africa	Cross-Sectional	283
2016	Rodriguez-Fernandez, R	[90]	Indonesia	Oceania	Cross-Sectional	22,550
2016	Gleason, R	[91]	Ethiopia	Africa	Cross-Sectional	231
2016	Sarfo, F	[92]	Ghana	Africa	Cross-Sectional	701
2016	Siedner, M	[93]	Uganda	Africa	Cohort	105
2016	Yen, YF	[43]	Taiwan	Asia	Cohort	22,581
2017	Benjamin, L	[40]	Malawi	Africa	Cohort	171
2017	Cumming, K	[94]	Thailand	Asia	Case–Control	610,688
2017	Mosepele, M	[95]	Botswana	Africa	Cross-Sectional	432
2017	Mosepele, M	[96]	Botswana	Africa	Cross-Sectional	208
2017	Siwamogsatham, S	[97]	Thailand	Asia	Cross-Sectional	316
2017	Sharma, SR	[98]	India	Asia	Cohort	91
2017	Feinstein, MJ	[99]	Uganda	Africa	Case–Control	205
2017	Kaseke, F	[100]	Zimbabwe	Africa	Cohort	450
2018	Salmazo, P	[101]	Brazil	South America	Case–Control	543
2018	Chow, F	[44]	China	Asia	Cross-Sectional	46
2018	Ekrikpo, U	[102]	Nigeria	Africa	Cross-Sectional	12,167
2018	Nonterah, E	[103]	Multiple	Africa	Cross-Sectional	8,872
2018	Lai, YJ	[104]	Taiwan	Asia	Cohort	26,272
2018	Bergman	[105]	India	Asia	Cross-Sectional	119
2018	Ellis, J	[33]	Malawi	Africa	Case Report	1
2018	Kamtchum-Tatuene, J	[106]	Malawi	Africa	Case–Control	139
2019	Hiransuthikul, A	[21]	Thailand	Asia	Cohort	50
2019	Juma, K	[35]	Kenya	Africa	Cross-Sectional	1,510
2019	Lin, H	[25]	Taiwan	Asia	Cohort	29,805
2019	Kamtchum-Tatuene	[31]	Malawi	Africa	Cross-Sectional	229
2019	Kiragga	[107]	Uganda	Africa	Cross-Sectional	559
2019	Aurpibul, L	[108]	Thailand	Asia	Cross-Sectional	155
2019	Mapoure Njankouo, Y	[109]	Cameroon	Africa	Cohort	608
2019	Brites, C	[26]	Brazil	South America	Cross-Sectional	451
2020	Yang, I	[110]	Uganda	Africa	Cross-Sectional	309
2020	Belisário, AR	[111]	Brazil	South America	Case–Control	83
2020	Wu, L	[42]	China	Asia	Cohort	128
2020	Matuja, SS	[112]	Tanzania	Africa	Cohort	369
2021	Kuate, LM	[38]	Cameroon	Africa	Cross-Sectional	43
2021	Vos, AG	[113]	South Africa	Africa	Cross-Sectional	289
2021	Kroon, L	[32]	South Africa	Africa	Cross-Sectional	140
2021	Zimba, S	[37]	Zambia	Africa	Cross-Sectional	272
2021	Siedner, MJ	[114]	Uganda	Africa	Cohort	309
2021	Osaigbovo, GO	[115]	Nigeria	Africa	Cross-Sectional	246
2021	Hiransuthikul, A	[116]	Thailand	Asia	Cross-Sectional	50
2021	Sarfo, FS	[46]	Ghana	Africa	Cross-Sectional	255
2021	Dirajlal-Fargo, S	[117]	Uganda	Africa	Cross-Sectional	40
2021	Nutakki, A	[118]	Zambia	Africa	Cross-Sectional	324
2021	Ounjaijean, S	[34]	Thailand	Asia	Cross-Sectional	60

**Fig. 2:**
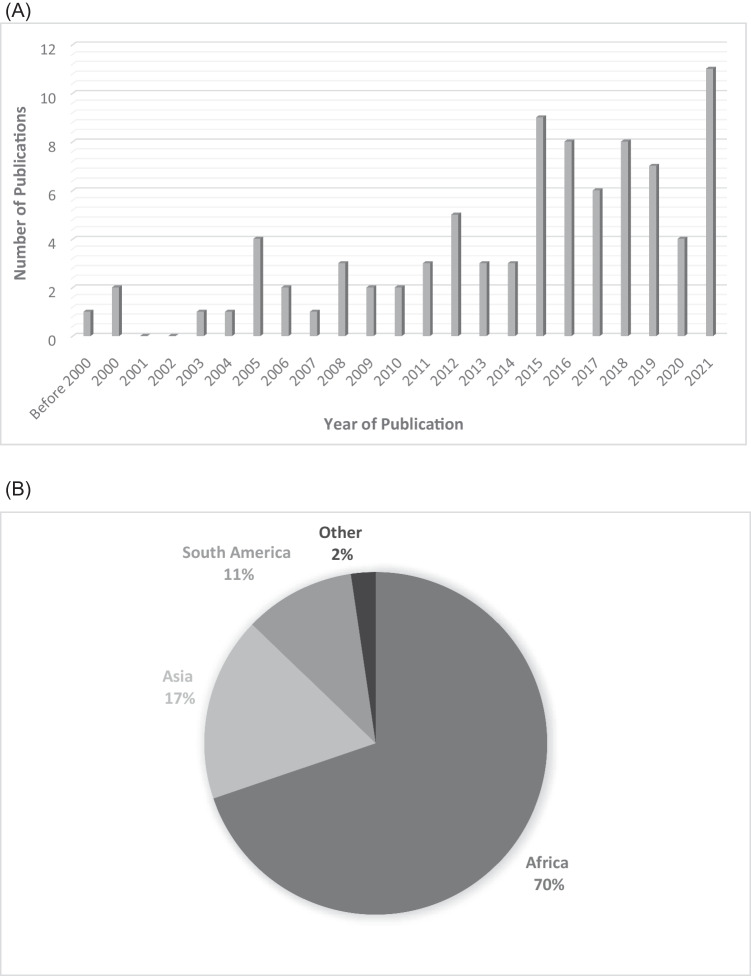

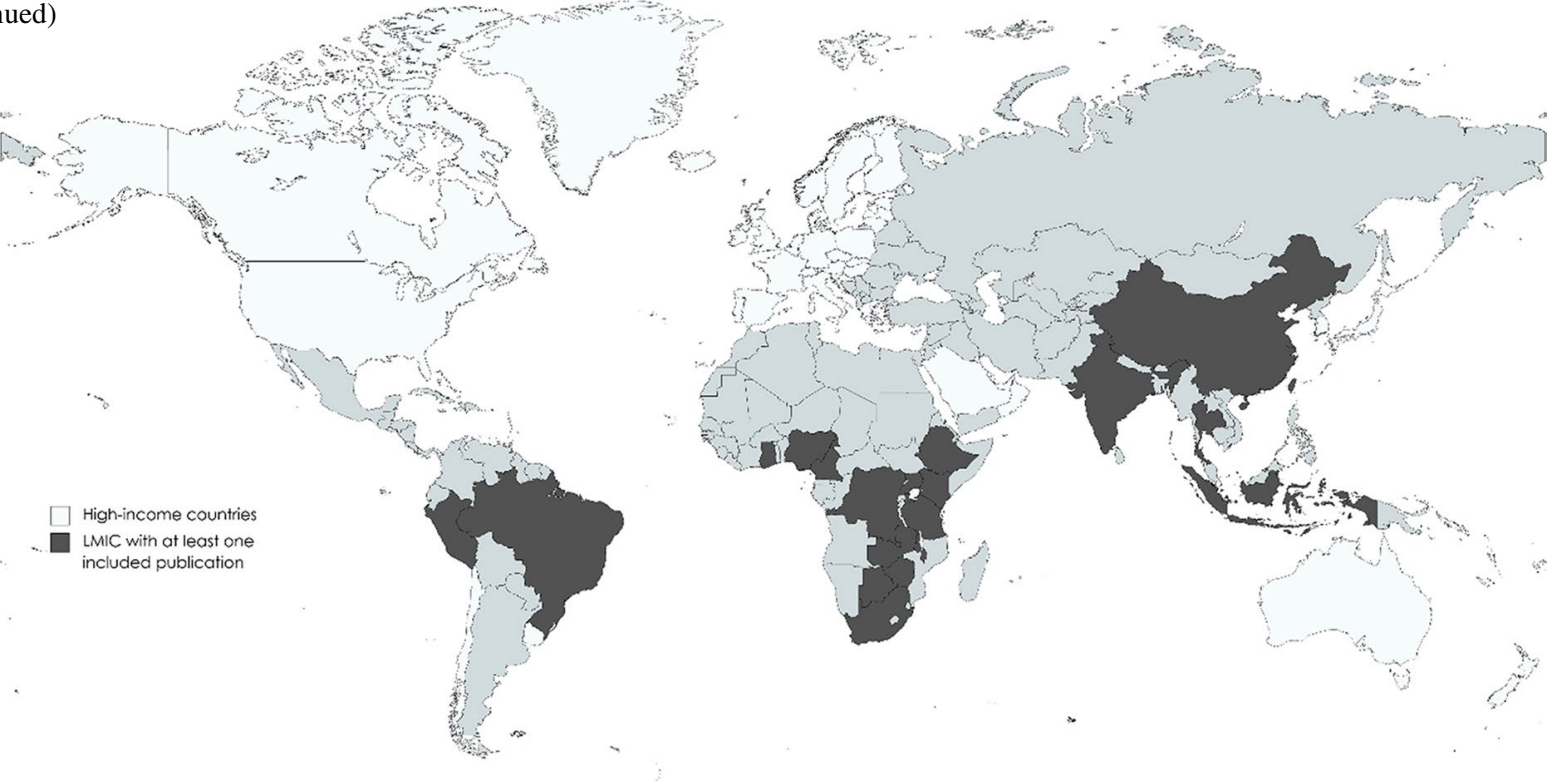
Distribution of included studies by year of publication (**A**), continent of origin (**B**) and country of origin (**C**)

The distribution of included publications by year (Fig. 2A) shows a clear trend toward increasing numbers of publications with time, with a marked increase in the number of publications per year beginning in 2015. This trend has largely been sustained through 2021, with more than five included publications each year since 2015. The largest number of included publications in any year was 11 in 2021. The majority of publications were from Africa (*n* = 61, 69%) followed by Asia (*n* = 17, 19%) and South America (*n* = 10, 11%) (Fig. 2B). Figure 2C shows countries where at least one article was included within LMICs (in black). Of 142 LMICs, only 20 countries (14%) are represented in the included publications.

Participants in the three included case series were people with HIV who experienced strokes in South Africa (*n* = 2) and Kenya [16••, 17, 18]. Participants in these studies had a mean age in the mid-thirties (32 to 36 years), and the majority had sub-optimally controlled HIV infection. One case series focused on identifying stroke aetiology and found high rates of stroke due to coagulopathies (49%) and meningitis (25%), with the remaining due to cardioembolism (9%) and hypertension (6%) [16••]. The outstanding two case series focused on HIV-associated vasculopathy as a cause of stroke. They found that HIV-associated vasculopathy primarily occurred in PLWH with low CD4 counts and was almost equally likely to present as an occlusive disease (51%) versus aneurysmal disease (49%) [17, 18].

### Epidemiology and Clinical Characteristics

#### Demographics and Epidemiology

Multiple studies described demographic and clinical data specifically for PLWH presenting with CVD, often comparing this with PLWH without CVD, or with HIV-negative CVD sufferers. Three studies reported cognitive function [19, 20••, 21]; the remainder focused on stroke (Table 2). Among these, the mean age of study participants ranged from 29 to 55 years. Between 1990 and 2010, the age range was 29–39 years, but increased to 40–55 years after 2010. Sex distribution across studies was variable, ranging from 9 to 75% female. Between 1990 and 2010, this was 41–75%, and 9–75% after 2010. All studies were hospital-based; four included community and outpatient clinic participants.

**Table 2:** Demographic, cerebrovascular disease phenotype and HIV factors in PLWH and cerebrovascular disease across the decades in LMICs

Author and year	Country	Setting	Age (yrs)	Female (%)	CD4 count cells/mm^3^	ART use (%)	Co-morbidities (%)	Prevalence of Surrogate markers of CVD	Prevalence of HIV in patients with CVD (%)
Hoffman 2000	S. Africa	Hospital- inpatient	29*	41	-	ND	ND	Carotid occlusions/low velocity: 24%	-
Mochan 2003	S. Africa	Hospital- inpatient	32*	60	< 200 in 40% 200–500 in 37% > 500 in 23%	0	HTN 6 AF 0 PFO 0 Meningitis 25 Coagulopathy 49	Carotid plaque: 0%	-
Connor 2004	S. Africa	Community	25–34**	52.7	-	-	-	-	1.94
Deshpande 2005	India	Combination Hospital inpatient / outpatient	15–45 = 75% 46–60 = 25%	ND	< 100 in 5% 100–200 in 40% 200–500 in 50% > 500 in 5%	0	ND	ND	-
Kumwenda 2005	Malawi	Hospital- inpatient	38*	75	AIDS in 53%	ND	HTN 11 DM 2 Smokers 7 Alcohol 6	Carotid plaque: 0%	48
Patel 2005	S. Africa	Hospital- inpatient	ND	51.8	-	ND	HTN 10.7 DM 1.8 Smoking 12.5 Alcohol 5.4	ND	19
Jowi 2007	Kenya	Hospital- inpatient	39*	42.1	120*	72	ND	ND	
Tipping 2007	S. Africa	Hospital- inpatient	33*	ND	< 200 in 46% > 200 in 54%	12	HTN 10 DM 0 Smoking 27 Alcohol 28 Co-infection 37	Intracranial vasculopathy: 20% Extracranial vasculopathy: 11%	6
Jowi 2008	India	Hospital Inpatient	-	-	$-$	$-$	-	-	2.5
Onwuchekwa 2008	Nigeria	Hospital inpatient	-	-	-	-	-	-	7.4
Benjamin Longo-Mbenza 2011	DRC	Hospital inpatient	43	54	107	-	HTN 100 DM 94 Smoking 100 Obesity 94 Excess alcohol 94	ND	15
Heikinheimo 2012	Malawi	Combination Hospital Inpatient / Outpatient	40*	52.0	WHO Stage 1/2 in 78%	22	HTN 24 DM 6 Dyslipidemia 14 Smoking 14	ND	34
Lee 2012	Thailand	Hospital- inpatient	51*	19	287*	48.6	HTN: 18.9 DM: 5.4 Dyslipidemia: 21.6 Smoking: 37.8 AF: 5.4 IVDU: 10.8 TB meningitis: 10.8 Cryptococcal meningitis:2.7	ND	-
Cumming 2013	Thailand	Hospital- Inpatient	41*	30.8	ND	ND	HTN: 9 DM: 3.3 Anaemia: 12.9	ND	0.14
Benjamin 2016#	Malawi	Hospital- inpatient and community	40**	55	-	40	HTN 42 DM 3 Smoking 9 Alcohol 21	Intracranial and extracranial vasculopathy:38%	37
Benjamin 2017#	Malawi	Hospital- inpatient	40**	55	190**	47	HTN42 DM3 Smoking 9 Alcohol 21	ND	31
Zimba 2017	Zambia	CombinationHospital inpatient / Outpatient	52*	56	431*	69	HTN:50 DM: 15 Smoking: 4 Alcohol: 19 Obesity: 4	ND	-
Kamtchum-Tatuene, 2018#	Malawi	Combination Hospital Inpatient / Outpatient	40**	50.0	136**	39.6	HTN:39.6 DM: 2.1 Dyslipidemia: 14.6 Smoking: 14.6 Alcohol: 21.2	ND	-
Chow 2018	China	Outpatient	41	13	467*	-	HTN < 10 Dyslipidemia: 18 Smoking: 27 DM: < 10 Alcohol: > 25	ND	ND
Kamtchum-Tatuene, 2019#	Malawi	Hospital- inpatient	42**	52.6	260*	47.4	HTN: 63.2 DM: 0 Smoking: 5.3 Alcohol: 10.5	ND	31
Mapoure 2019	Cameroon	Hospital- inpatient	51**	35	351*	83	HTN: 65	ND	7
Hiransuthikul 2019	Thailand	Outpatient	55	39.5	616	-	-	-	-
Hiransuthikul 2021	Thailand	Hospital- inpatient	53**	22	295**	74	HTN: 52 Dyslipidemia: 62 Smoking: 55 DM: 28 Alcohol: 45	ND	1
Kroon 2021	South Africa	Hospital- inpatient	41*	52.9	< 250 in 68.8%	56.3	HTN: 81 Co-infection: 78 Dyslipidemia: 62	ND	23
Kuate 2021	Cameroon	Hospital- inpatient	52*	69.8	304*	58.1	HTN 72.1 DM 7 Smoking 34.9 Alcohol 48.8	Framingham Score: Low: 67.4% Intermediate/High: 32.6%	-
Zimba 2021	Zambia	Hospital- inpatient	48*	60	260**	83	HTN: 65 No CVD risk factors:34	ND	21

Abbreviations: *ND*, not documented; *NA*, not applicable; *HTN*, hypertension; *DM*, diabetes mellitus; *AF*, atrial fibrillation; *PFO*, patent foramen ovale; *TB*, tuberculosis; *NIHSS*, National Institutes of Health Stroke Scale – median score unless otherwise stated; *ART*, anti-retroviral therapy; *CVD* = cerebrovascular disease; *PLWHIV*, people living with HIV

*Mean of PLWH **median of PLWH #data extracted from the same cohort.$\mu$= PLWH specific data not listed. Studies included where cerebrovascular disease was a primary outcome

Twelve studies assessed the prevalence of HIV in patients presenting with CVD, while others (*n* = 4) examined the incidence or prevalence of CVD in patients with HIV. Reported incidence rates varied: in Cameroon, Mapoure et al. (2016) estimated a stroke rate of 3 per 1000 person-years; similarly in Taiwan a rate of 2.12 per 1000 person-years was found (Lin et al., 2019); while in Ghana, Sarfo et al. (2021) found a rate of 12.24 per 1000 person-years [22–25]. Examining stroke prevalence, Brites et al. found a rate of 4.4% among PLWH [26]. The overall hospital prevalence of HIV among stroke admissions in PLWH was between 0.14 and 48% when provided. In contrast, the prevalence of HIV in the CVD population ranged from 2.5 to 48% between 1990 and 2010 and 0.14–8% after 2010.

Our case–control study from Malawi (*n* = 723) compared hospital-based stroke cases with matched population-based non-stroke controls to estimate the risk of stroke; they found that HIV infection was associated with an increased odds of stroke (adjusted odds ratio [aOR] 3.28, CI [2.05–5.25]) [7]. Moreover, HIV accounted for the second-highest population attributable fraction (15%) overall and the highest among young populations (PAF 42%; age < 45 years) [7].

#### HIV Factors

The stage of HIV infection during hospital admission was variable across the three decades. The average CD4 T-cell count ranged from 190 to 431 cells/mm^3^. Between 1990 and 2010, three studies reported advanced disease (i.e. CD4 count < 200 cells/mm3) in 40–53% of cases [27–30], whereas after 2010, more studies had a median CD4 T-cell count above 200cells/mm^3^ (Table 2). Viral load was rarely described. Kamtchum-Tatuene et al. reported a median viral load of 1,884 copies/ml, with 55.6% of participants having > 1,000 copies/ml; while in a South African study, 62.5% of patients were not virologically suppressed, despite many patients reporting being on ART [31, 32]. The use of ART was well described, and as shown in Table 2, coverage was generally poor (i.e. < 12%) between 1990 and 2010. After 2010, the ART prevalence substantially increased (22–83%), coinciding with the roll-out of ART programs in these regions.

Only a few studies discussed ART and stroke risk. One of which was ours, we identified an increased stroke risk early in the use of treatment, possibly suggesting an Immune Reconstitution Inflammatory Syndrome (IRIS)-related mechanism [7]. Furthermore, we showed no risk with long-term ART use, and speculated that this might have been underpowered or affected by competing risks. One case report described an ischaemic stroke as a paradoxical IRIS reaction [33]. Some studies corroborated previous findings that protease inhibitors (PIs) increase rates of dyslipidaemia, and Juma et al. found that nucleotide reverse transcriptase inhibitor (NRTI)-based regimens were associated with raised total cholesterol [34, 35].

#### Cerebrovascular Disease (CVD) Risk Factors

Multiple authors examined traditional cardiovascular risk factors in PLWH. High rates of hypertension were described in many of these studies, particularly those completed more recently. Hypertension prevalence between 1990 and 2010 was 6–11%, and after 2010, this increased to 10–72%. However, studies comparing PLWH to aged-matched HIV-negative stroke patients generally showed no significant difference between the most common cardiovascular risk factors, including hypertension, diabetes mellitus and dyslipidaemia [7, 36], while one study from Zambia found significantly lower rates of traditional CVD risk factors among PLWH compared to HIV-uninfected adults with stroke [37].

Scoring systems for CVD risk were infrequently assessed in studies of stroke. Kuate et al. found that the Framingham score correlated poorly with stroke risk in PLWH, with 67.4% of patients given a low-risk score, likely underestimating overall risk [38].

Carotid disease is a surrogate of CVD. Although there was heterogeneity regarding the definition of carotid disease across studies, those limited to an extracranial evaluation reported a prevalence of carotid disease ranging between 0 and 24%.

## Aetiology and Outcome in PLWH and Cerebrovascular Disease

### Aetiology

Eight out of 53 (15%) eligible articles reported on the aetiology and/or stroke outcome in PLWH. Prevalence of CT or MRI brain imaging performed in a selected or unselected cohort varied from 87 to 100%. This high uptake was consistent across two decades. The prevalence of ischaemic stroke was higher (57–96%) compared with intracerebral haemorrhage (4–33%; Table 3). The TOAST classification and its variations were used to provide a template to describe the aetiology. Crucially, less than the minimum set of investigations, as agreed in a consensus statement on HIV and stroke, were performed, thus precluding accurate attribution of stroke aetiology [39]. For example, approximately 60% of study participants had CSF to investigate opportunistic infections, and only 60% had an electrocardiogram or echocardiogram looking for a cardioembolic source. Common aetiologies included large vessel vasculopathy (20–37%) and opportunistic infections (5–37%). Twenty per cent of HIV-associated ischaemic stroke was attributed to opportunistic infection in one study [40]. Likewise, Tipping et al. found that a third of PLWH with stroke had evidence of intercurrent opportunistic infections [29]. These study populations had low CD4 T-cell count [29, 40]. In other studies, tuberculous and cryptococcal meningitis and CMV encephalitis were associated with increased stroke risk. Cardioembolic stroke was low in frequency (6–8%) [41–43].

**Table 3:** Aetiology and outcome in PLWH and cerebrovascular disease across the decades in LMICs

Author and year	Country	Ref no.	*N*	Investigations available to assess aetiology	Type of cerebrovascular disease and proportion	Aetiology of arterial ischaemic stroke in PLWH	Outcome
Hoffmann 2000	South Africa	[20]	22 HIV + cases and 22 HIV- controls	CT (9%) or MRI (91%), doppler ultrasound 95%, cerebral angiography 45%, Echo 95%, CSF studies 67% (glucose, protein, pleocytosis and cryptococcal antigen mentioned)	Among cases: Arterial ischaemic 95% Venous ischaemic 5%	Large vessel vasculopathy 36% Vasculitis 0% Small vessel disease 0% Coagulopathy 0% Opportunistic infection (CNS cryptococcal infection) 5% cardioembolic 9% Other: unknown 50%	MRS: No significant difference between the two groups Cognitive function: HIV group: larger scale network impairment such as frontal system syndromes (4/22; 18%) and aphasia (10/22; 45%) compared to the control group (frontal system syndrome 3/22; 14% and aphasia 8/22; 36%)
Mochan 2003	South Africa	[16]	35 HIV +	CT (100%), doppler ultrasound 100%, cerebral angiography 63%, ECG 94% Echo 94%, CSF studies 94% (protein, glucose, cell count, VZV + CMV + HSV PCR, cryptococcal antigen test), CXR 0%, CD4 counts 100%, coagulation panel 100% and autoimmune screen (anticardiolipin antibodies) 100%	Arterial ischaemic 94% Intracerebral haemorrhage 6%	Large vessel vasculopathy 6% Vasculitis 3% Small vessel disease 6% Coagulopathy 49% Opportunistic infection (3 tuberculous, 1 pyogenic, and 4 viral) 25% Other (cardioembolic) 9% No potential cause 14%	ND
Tipping 2007	South Africa	[29]	61 HIV + vs. 205 HIV-	CT or MRI (100%), doppler ultrasound 0%, cerebral angiography 0%, CD4 counts 72%, coagulation panel 0% and autoimmune screen 0%	Among HIV + cases: - Arterial ischaemic 96% - Intracerebral haemorrhage 4%	Large vessel vasculopathy 20% Vasculitis 5% Small vessel disease 0% Coagulopathy 19% Opportunistic infection (specify) 37%; tuberculosis (16 patients), varicella zoster (three patients), pneumocystis pneumonia (three patients), cryptococcal meningitis (two patients) and Kaposi's sarcoma (one patient) Other (specify) 0%	MRS: 4.0 in the HIV positive group and 4.2 in the HIV uninfected group (p = 0.70). For the other inpatient post-stroke complications, including death, there was no difference between HIV positive and uninfected patients
Heikinheimo 2012	Malawi	[36]	50 HIV + vs. 84 HIV -	CT or MRI (87%), doppler ultrasound 0%, cerebral angiography 0%, ECG 14% Echo 0%, CSF studies 0%, CXR 0%, CD4 counts 80%, Syphilis RPR coagulation panel 0% and autoimmune screen 0%	Arterial ischaemic stroke: 40 (80%) Intracerebral haemorrhage: 5 (10%)	Cardioembolic (atrial fibrillation); 4/50 (8%) (only 7 HIV patients had an ECG, so it could be an underestimation)	6 weeks: Death; HIV- 23%, HIV + 18%, mRS 4–5; HIV- 32.1 HIV + 14% (*p* = 0.015) In the multiple logistic regression these variables lost their significance Death at 6 months: HIV- 8%, HIV + 16%, mRS 4–5; HIV- 11.9 HIV + 6% (*p* = 0.58) Death at 1-year: HIV- 6%, HIV + 2%, mRS 4–5; HIV- 12% HIV + 2% (*p* = 0.29)
Benjamin 2017	Malawi	[40]	64 HIV + vs. 107 HIV-	MRI 100%, doppler ultrasound 100%, cerebral angiography 0%, CSF studies 100%, = CD4 counts 100%, coagulation panel 88% and autoimmune screen (anticardiolipin)antibodies lupus anticoagulant, anti-β2 –glycoprotein 1 100%	Arterial ischaemic 100% for both cases and controls	Large vessel vasculopathy 21% Vasculitis 14% Small vessel disease 2% Coagulopathy (antiphospholipid syndrome) 9% Opportunistic infection (VZV, MTB, Syphilis) 25% Cardioembolism 6% Other: Cryptogenic stroke 17% Multifactorial 2% Inconclusive 3%	NIHSS: 12 (8–14) in HIV + and 11 (7–18) in HIV-; not significant Hospital mortality: 11 (17) in HIV + and 10 (9) in HIV-. Not significant
Zimba 2017	Zambia	[82]	52 HIV + vs. 52 HIV-	CT or MRI (100%), CD4 counts 100%, coagulation panel 100%	Arterial infarct 100% for both cases and controls	Large Vessel vasculopathy 31% Vasculitis 0% Small Vessel disease 15% Coagulopathy 13% Opportunistic infection 0% Cardioembolic 4% Unknown 10%	NIHSS: 8 (2 – 15) in HIV + compared to 11 (4 – 19) in HIV-: *p* = 0.25
Hiransuthikul 2019	Thailand	[116]	50 HIV +	Not specified	Ischaemic 100%	Large Vessel vasculopathy 8% Vasculitis 0% Small Vessel disease 48% Other determined 14% Unknown 26%	
Kuate 2021	Cameroon	[38]	43 HIV +	CT or MRI (100%), CD4 counts 100%, coagulation panel 100%	Ischaemic Stroke 84% Intracerebral haemorrhage 16.3%	Opportunistic infection 28 (65.1%)	NIHSS > 15 7 (20%) Death < 7 days 14% Death < 1 month 35% Death < 1-year 47%
Zimba 2021	Zambia	[37]	58 HIV +	CT/MRI (91%)	Ischaemic Stroke 57% Intracerebral haemorrhage 33%	ND	In hospital mortality: HIV + 12 (21%), HIV- 50 (23%) *p* = 0.65

### Outcome

Six studies reported on hospital mortality and showed high rates (17–21%) Table 3 [20, 29, 37, 38, 40]. However, Heikinheimo et al. showed that death at 6 weeks was higher in the HIV-negative adults with stroke [HIV-negative mortality: 23%, mortality in PLWH and stroke: 18%], but when accounting for age, there was no significant difference in mortality [36]. Furthermore, the functional outcome at 6 weeks was significantly better in PLWH [mRS of 4–5: 32% of HIV-negative and 14% of PLWH (*p* = 0.015)]. Risk factors for increased mortality included low GCS on admission (*p* = 0.046), fever during hospitalization (*p* = 0.003) and hypertension (*p* = 0.04) [37].

There were limited reports on cognitive outcome, one study described cognitive function and demonstrated the involvement of frontal system syndromes (4/22; 18%) and aphasia (10/22; 45%) compared to the control group (frontal system syndrome 3/22 (14%) and aphasia 8/22(36%)), and another looked at cerebral vasoreactivity in PLWH and correlated the presence of this with good cognitive performance [20, 44]. Another showed no association using the Montreal Cognitive assessment, and cognitive performance and surrogate markers of CVD [21].

NIHSS scores, a marker of stroke severity, were reported infrequently. Most studies showed unadjusted scores of minor/moderate severity (NIHSS < 14). Scores were generally lower than those reported in HIV-negative control groups, indicating lesser stroke severity in PLWH. Our study from Malawi showed a median score of 12 in PLWH and 13.5 in HIV-negative cases [31]. Likewise, Kroon et al. showed a mean of 11 in PLWH and 14 in those HIV-negative individuals with stroke [32].

## Discussion

Our systematic review found that the CVD landscape in PLWH residing in LMICs between 1990 and 2021 has evolved. Due to the success of ART uptake, PLWH are living longer. We found that among those manifesting with CVD, the median age has increased, and a greater number of patients are on ART in more contemporary cohorts. In turn, the CD4 + T-cell count has also increased over time. Moreover, the burden of overlapping CVD risk factors, notably hypertension, also increased. Mortality rates across the decades remained high but appeared not to differ compared to the HIV-negative populations. The numbers of studies published annually increased over the last 5 years in keeping with reports of an increasing burden of CVD in PLWH [1].

The top ten countries with the highest prevalence of HIV, ranging from 10–27%, were found in sub-Saharan Africa (SSA). Haiti is the LMIC outside of SSA with the highest HIV prevalence (1.9%) and has the 24^th^ highest HIV prevalence globally [45]. In this review, the representation of the publications emerging from LMICs was a fair reflection of where HIV is most burdensome as 69% of included studies were from SSA. However, absolute numbers of publications were low overall, highlighting the limited data on HIV-associated CVD from LMICs.

Only a handful of studies in the last 5-years estimated a stroke burden, and these ranged from 3 to 12 per 1000 person-years [24, 25, 46]. The under-representation of stroke burden assessments in LMICs, especially SSA, has been a historical problem but improving [8]. With an ageing population and an overlapping burden of CVD risk factors in PLWH, we would have expected an increased number of those with neurocognitive impairment as well, but this was rarely reported. The reason is likely multifactorial, including (1) inconsistent coding; in 2017, cerebrovascular disease was coded separately from cardiovascular diseases in the ICD-11 classification; prior to this change, it is likely to have been underestimated, (2) challenges with cross-culture bias in neuropsychology testing tools, (3) an underrepresentation of neuroscience researchers in LMICs, who would typically undertake these studies [47–49]. A degree of education is often assumed with the tools needed to assess neurocognition and thus risks overestimating the burden, but this is not the current problem. Rather, a near complete absence of data on CVD contributions to neurocognitive impairment among PLWH residing in LMICs was noted. These limitations extend beyond HIV infection to data on the burden of dementia in LMICs and need addressing [50].

Notably, most studies had brain imaging to define a stroke and determine the stroke type. Ischaemic stroke was the predominant type of stroke, accounting for a prevalence > 85%; this is consistent with studies from HICs [4]. However, in approximately 40% of studies focused on aetiology, there was a preferential selection for ischaemic stroke type, underrepresenting HIV-associated intracerebral haemorrhagic stroke and limiting our ability to corroborate findings of an associated risk as described in HICs [4]. Beyond brain imaging, there was variability in the panel used to investigate CVD and define the aetiology. For example, treatable aetiologies such as opportunistic infections and cardioembolism, determined by CSF analysis and electrocardiogram/cardiac echocardiogram, respectively, were only performed in 60% of studies. A consensus approach to identifying stroke aetiology among PLWH, led by some of the authors, was published in 2017, where a minimum battery of tests was proposed to define the common aetiologies found in PLWH and presenting with CVD [39]. However, resource limitations result in difficulty obtaining even this limited battery of investigations in many LMIC settings, as does human resource limitations as neurologists and other stroke experts are often lacking in these settings.

Unsurprisingly, the outcome of CVD among PLWH remains poor. Although poorer health systems may play a part, the failure to systematically screen and manage treatable aetiologies may also be relevant. Additionally, the reporting of outcome measures was variable in terms of measures used and the timing of when events were measured. Guidance on standard reporting of outcome measures in stroke has been proposed and could be applied to CVD studies in PLWH [51, 52].

The striking rise in hypertension between 1990 and 2021 was apparent in our review. Already, policy implementation exists in how to reduce the burden of hypertension in PLWH (primary prevention), primarily by exploiting well-developed HIV health care systems [53]. However, the challenge with polypharmacy and drug interactions poses a different barrier in LMICs. Particular attention should be paid to secondary prevention as these individuals with an accrued disability may encounter additional barriers to accessing health systems and inadvertently be neglected.

A major limitation to this review is the dependence on mostly low-level observational studies, which limited our ability to pool data in a meta-analysis, and was subject to bias and confounding. Moreover, almost all studies were hospital-based, restricting our understanding of milder CVD cases at risk of subsequent events and further disability or fatal events in the community. Although three studies demonstrated excess CVD risk in PLWH, which is consistent with reports in HICs, we still have less understanding of CVD-associated cognitive risk in PLWH. Current data suggest that some of the underlying pathobiology of stroke and vascular-associated cognitive impairment might be interrelated [54]. Therefore, it is essential to develop a robust understanding of any vascular component involved in cognitive impairment in PLWH so that successful primary prevention strategies for stroke can be integrated with those for cognitive impairment. Investment in surveillance cohorts of PLWH across SSA focusing on non-communicable diseases is emerging, but more are needed. In time, this will give robust incidence, prevalence, mortality and disability metrics and inform policy [53, 55, 56]. However, standardised reporting of risk factors, aetiology and outcome will be crucial in supporting the advancement of this field.

## Conclusions

The clinical phenotype of CVD among PLWH over the last three decades in LMICs has evolved and transitioned to an older group with overlapping cerebrovascular risk factors. There is an important need for further rigorous population-based studies and large observational cohort studies of PLWH in LMICs and to standardise reporting to facilitate understanding, guide appropriate interventions and evaluate its impact.
